# Microbe-Responsive Proteomes During Plant–Microbe Interactions Between Rice Genotypes and the Multifunctional *Methylobacterium oryzae* CBMB20

**DOI:** 10.1186/s12284-023-00639-y

**Published:** 2023-05-05

**Authors:** Denver I. Walitang, Aritra Roy Choudhury, Parthiban Subramanian, Yi Lee, Geon Choi, Kun Cho, Sung Ho Yun, Aysha Rizwana Jamal, Sun-Hee Woo, Tongmin Sa

**Affiliations:** 1 Department of Environmental and Biological Chemistry, Chungbuk National University, 28644 Cheongju, Republic of Korea; 2 College of Agriculture, Fisheries and Forestry, Romblon State University, Romblon, Philippines; 3 Microbiome Network and Department of Agricultural Biology, Colorado State University, Fort Collins, CO USA; 4 National Agrobiodiversity Center, National Institute of Agricultural Sciences, Rural Development Administration, Jeonju-si, Republic of Korea; 5 PG and Research Department of Biotechnology and Microbiology, National College, Tiruchirapalli, Tamilnadu India; 6 Department of Industrial Plant Science and Technology, Chungbuk National University, 28644 Cheongju, Republic of Korea; 7 Bio-chemical Analysis Team, Center for Research Equipment, Korea Basic Science Institute, Cheongju, Republic of Korea; 8 Department of Agronomy, Chungbuk National University, Cheongju, Republic of Korea; 9 The Korean Academy of Science and Technology, Seongnam, Republic of Korea

**Keywords:** Proteomics, LC–MS/MS, Plant growth promotion, *Methylobacterium*, Rice

## Abstract

**Background:**

Rice is colonized by plant growth promoting bacteria such as *Methylobacterium* leading to mutually beneficial plant–microbe interactions. As modulators of the rice developmental process, *Methylobacterium* influences seed germination, growth, health, and development. However, little is known about the complex molecular responsive mechanisms modulating microbe-driven rice development. The application of proteomics to rice-microbe interactions helps us elucidate dynamic proteomic responses mediating this association.

**Results:**

In this study, a total of 3908 proteins were detected across all treatments of which the non-inoculated IR29 and FL478 share up to 88% similar proteins. However, intrinsic differences appear in IR29 and FL478 as evident in the differentially abundant proteins (DAPs) and their associated gene ontology terms (GO). Successful colonization of *M. oryzae* CBMB20 in rice resulted to dynamic shifts in proteomes of both IR29 and FL478. The GO terms of DAPs for biological process in IR29 shifts in abundance from response to stimulus, cellular amino acid metabolic process, regulation of biological process and translation to cofactor metabolic process (6.31%), translation (5.41%) and photosynthesis (5.41%). FL478 showed a different shift from translation-related to response to stimulus (9%) and organic acid metabolic acid (8%). Both rice genotypes also showed a diversification of GO terms due to the inoculation of *M. oryzae* CBMB20. Specific proteins such as peptidyl-prolyl cis–trans isomerase (A2WJU9), thiamine thiazole synthase (A2YM28), and alanine—tRNA ligase (B8B4H5) upregulated in IR29 and FL478 indicate key mechanisms of *M. oryzae* CBMB20 mediated plant growth promotion in rice.

**Conclusions:**

Interaction of *Methylobacterium oryzae* CBMB20 to rice results in a dynamic, similar, and plant genotype-specific proteomic changes supporting associated growth and development. The multifaceted CBMB20 expands the gene ontology terms and increases the abundance of proteins associated with photosynthesis, diverse metabolic processes, protein synthesis and cell differentiation and fate potentially attributed to the growth and development of the host plant. The specific proteins and their functional relevance help us understand how CBMB20 mediate growth and development in their host under normal conditions and potentially link subsequent responses when the host plants are exposed to biotic and abiotic stresses.

**Supplementary Information:**

The online version contains supplementary material available at 10.1186/s12284-023-00639-y.

## Background

Plants are continually associated with microorganisms throughout their lifecycle with their associations established at the seed level even before they begin to germinate. These microbes are major factors that drive growth, intermittent or continuous stress tolerance, and overall plant productivity which works vice versa as plants also influence their associated microbial community and function (Chialva et al. 2022). Rice plants have been found to establish intricate relationships with beneficial microbes from the soil and their phyllosphere forming endophytic plant–microbe associations that lead to enhanced plant growth and development (Sessitch et al. 2012; Hardoim et al. 2011, 2012). In many cases, beneficial plant–microbe interactions also lead to enhanced plant fitness and protection under biotic and abiotic environmental stresses (Pieterse et al. 2014; Balmer et al. 2015). Studies on the interactions of rice with bacterial endophytes also reveal complex microbial communities (Sun et al. 2008; Hardoim et al. 2011, 2012; Walitang et al. 2018), the functional properties of these microbial communities as well as their interaction mechanisms with rice (Sessitch et al. 2012; Walitang et al. 2017).

Increasing studies on rice microbiome and rice-microbe interactions show the potential impact of rice-associated microbiomes affecting the growth and development of rice under normal and stress conditions. On the other hand, rice across different genotypes and cultivars intrinsically has differences in its natural tolerance to stress. Rice is generally very sensitive to moderately sensitive to salt stress, but some landraces and cultivars have attained a certain degree of salt tolerance (Gregorio et al. 1997). The rice genotypes IR29 and FL478 are commonly used salt-sensitive and salt-tolerant controls, respectively (Walitang et al. 2019), and that distinct phenotypic and physiological differences, especially under salt stress are evident between these genotypes (Hosseini et al. 2015; Senadheera et al. 2012; Mirdar Mansuri et al. 2019). Chatterjee et al. (2019) have shown the enhanced salt tolerance of IR29 and FL478 due to inoculation of *Methylobacterium oryzae* CBMB20. However, we still need to establish system-wide proteomic changes occurring when *M. oryzae* CBMB20 is used as a bioinoculant in these genotypes under non-stress conditions and the potential linkages of their responses to subsequent changes of proteomes meditated by *M. oryzae* CBMB20 under salt stress conditions.

Elucidating and understanding these complex plant–microbe interactions, through omics studies, provide potential avenues to improve rice productivity while supporting sustainable agriculture and environmental resilience. In addition, determinants of successful and beneficial plant–microbe interactions greatly depend on the host plant, the microbial nature, and the environmental conditions. The host genotype generally dictates the recruitment of microbial inhabitants and the plant response to endophytes are conditioned by the plant genotype (Rosenblueth and Martinez-Romero 2006; Hardoim et al. 2011; Johnston-Monje and Raizada 2011). On the other hand, competent bacterial endophytes also possess adaptations that allow long-term persistence, particularly through proteins and metabolites that promote plant growth and health (Hardoim et al. 2008, 2015). In rice plants, these endophytes can transfer across generations through their seeds and majorly contribute to a plant’s disease resistance (Walitang et al. 2019; Matsumoto et al. 2021).

Among the rice-associated microbial communities, the genus *Methylobacterium* is a multifaceted, PGPB microbial group. *Methylobacterium* spp. are potentially ubiquitous to all plants and are substantially involved in some plant biological processes (Holland and Polacco 1994). The strain *Methylobacterium oryzae* CBMB20 also exemplifies this group. CBMB20 was isolated in the stem endosphere of *Oryza sativa* subsp. *japonica* cv. Nam-peong (Madhaiyan et al. 2004; Madhaiyan 2007a). From its initial detection as a potential diazotroph, CBMB20 was then used and proven to promote growth in multiple plant hosts including other *Oryza sativa japonica* cultivars (Lee et al. 2006; Madhaiyan et al. 2010; Roy Choudhury et al. 2022), *Oryza sativa indica* cultivars (Chatterjee et al. 2019), red pepper (Ryu et al. 2006; Madhaiyan et al. 2010; Lee et al. 2011), canola (Madhaiyan et al. 2007b), soybean (Subramanian et al. 2015), and tomato (Ryu et al. 2006; Indiragandhi et al. 2008; Madhaiyan et al. 2010; Yim et al. 2013; Yim et al. 2014; Chanratana et al. 2018; Chanratana 2019). Inoculated CBMB20 could persist in the rhizosphere and phyllosphere (Lee et al. 2011; Madhaiyan et al. 2010), the spermosphere (Chanratana et al. 2018), and the endosphere (Lee et al. 2011; Yim et al. 2013, 2014; Walitang et al. 2023).

*Methylobacterium oryzae* CBMB20 ability to promote plant growth in multiple plant hosts could be attributed to its ability to produce indole acetic acid (IAA) and cytokinin (Lee et al. 2006; Ryu et al. 2006; Madhaiyan et al. 2007b). It is also involved with plant nutrient absorption and dynamics through thiosulfate oxidation (Anandham et al. 2007), potential nitrogenase, phosphatase, and urease activity (Madhaiyan et al. 2004, 2010). One of its most crucial attributes is the production and activity of 1-aminocyclopropane-1-carboxylate (ACC) deaminase enzyme. This enzyme modulates plant stress ethylene levels by catabolic hydrolysis of ACC, enhancing stress tolerance and restoring continued plant growth (Madhaiyan et al. 2007b; Indiragandhi et al. 2008; Yim et al. 2013; Yim et al. 2014; Chanratana 2018; Chatterjee et al. 2019; Roy Choudhury et al. 2022; Roy Choudhury et al. 2023). The whole genome sequencing of CBMB20 supports the multifaceted nature of this versatile PGPB. Compared to closely related *Methylobacterium* strains, CBMB20 has more genes encoding motility and signaling indicating its more efficient responses to environmental cues and phyllosphere/endosphere colonization. *M. oryzae* CBMB20 is also equipped with genes related to PGP including genes for producing auxin, cytokinin, ACC deaminase, PQQ, and vitamin B12, as well as those involved with nutrient mobilization such as genes encoding for phytase, C-P lyase system, sulfur-oxidation, urease, and their associated transporters (Kwak et al. 2014).

Many of the studies involving PGPB are conducted at the physiological and organismal levels involving selected parameters. However, plant–microbe interaction especially with a multifaceted PGPB involves dynamic and systemwide physiological changes mediated by transcripts, proteomes, and metabolites. Plant–microbe interaction is not a simple outcome of one-way interaction but rather multigenic/multilevel plant responses. Proteomic analyses in recent times have proved the beneficial role of endophytic bacteria in the activation of stress responses and survival mechanism of *Eichhornia* (Priya et al. 2022). Therefore, proteome analysis through high throughput sequencing, quantification, and identification involved in plant–microbe interactions can help us better understand the complex networks that mediate such interactions.

The current study evaluates proteins and proteomes of two rice genotypes, IR29 and FL478, and how their proteomes change due to inoculation of the multifaceted endophytic *Methylobacterium oryzae* CBMB20 identifying microbe-responsive proteins providing insights into the molecular mechanisms allowing PGPB to directly and indirectly promote plant growth. Upon successful identification, the proteome can provide valuable information towards understanding the complex and fine-tuned plant–microbe interactions in rice and will help link future plant responses such as cross-tolerance to different stress conditions mediated by *M. oryzae* CBMB20.

## Materials and Methods

### Bacterial Strain and Preparation of Inoculum

*Methylobacterium oryzae* CBMB20 was used as an inoculum for this study. The bacterial strain was grown in ammonium mineral salt (AMS) medium with 0.5% sodium succinate as a carbon source grown until OD_600_ ~ 0.8. The strain was harvested using 0.03 M MgSO_4_. For confocal microscopy, a previously tagged *M. oryzae* CBMB20-gfp (Kim et al., 2013) was used as an inoculant and grown and prepared in the same condition as the wild type except that the AMS medium was supplemented with 50 µg mL^−1^ kanamycin.

### Plant Material, Growth Conditions and Bacterial Inoculation

Rice (*Oryza sativa* L. subsp. *indica* cv. IR29; cv. FL478) seeds were obtained from the Rural Development Administration (RDA), Republic of Korea. Rice seeds were surface sterilized (Chatterjee et al. 2019) and germination was induced by soaking them in distilled water and then incubating them without light for 2 days. Bacterial inoculation and seedling transplantation were done according to Roy Choudhury et al. (2022). Germinated seeds were sown in nursery soil and grown in greenhouse conditions under natural illumination. Seedlings (7-day-old) were bacterized by dipping the roots in the inoculum suspension for 2 min, transplanted in seedling trays (individual hole diameters: 5 cm—outer diameter; 3 cm—inner diameter; 6 cm—depth) containing nursery soil and an additional 5 mL inoculum was added in the rhizosphere region. A solution of 0.03 M MgSO_4_ was used for non-inoculated plants. The same setup was made for rice plants inoculated with *M. oryzae* CBMB20-gfp to be used for confocal microscopy. The experiment was designed with four treatments namely, non-inoculated IR29 (IR29-NI), non-inoculated FL478 (FL478-NI), *M. oryzae* CBMB20 inoculated IR29 (IR29-I), and *M. oryzae* CBMB20 inoculated FL478 (FL478-I) and replications for each treatment.

### Confocal Microscopy

Freshly harvested 14-day-old IR29 and FL478 plant samples (roots, shoots, and stem) were removed from at least three seedling plants inoculated with *M. oryzae* CBMB20-gfp, washed with sterile distilled water and then blotted dry. Hand-cut transverse and cross sections of surface-sterilized roots, shoots, and stems were used to check the endophytic colonization by the inoculated strains. The samples were mounted using sterile glycerol under a coverslip. The samples were stored at 4 °C for 24 h prior to confocal microscopy. Microscopic observations were performed using Leica TCS SP2 confocal system (Leica Microsystems Heidelberg GmbH, Manheim, Germany) equipped with an Arion laser (gfp: excitation, 488 nm; emission filter BP500-530). Image acquisitions were carried out using a × 20 and × 50 objectives and processed with Zen software package (version 2.5.1227a).

### Protein Extraction, Purification and Digestion

Seedlings (14-day-old) of IR29 and FL478 were harvested containing 30 plants per replicate. Fresh leaf samples (0.5 g) were finely ground with liquid nitrogen and TCA (trichloroacetic acid)/Acetone precipitation method for protein extraction was done according to Roy et al. (2016). Bradford method (1976) was used to quantify the extracted total protein content. The methanol-chloroform method (Komatsu et al. 2013) was used to purify 150 µg protein samples which were alkylated with iodoacetamide before digesting with trypsin.

### LC–MS/MS Analysis

LC–MS/MS analysis of digested protein samples was done using an LTQ Orbitrap mass spectrometer (Thermo Fischer, Bremen, Germany) coupled with 1100 nano-flow HPLC system (Agilent) equipped with a nano electrospray ion source. This two-column system consists of an interconnected (three-way tee connector) pre-column, waste line, and the analytical column (C18 AQ, 3 μm, 100 μm × 15 cm, NanoLC, USA). Aliquots of the peptide samples (10 μL) were loaded with an autosampler into the C18 trap column (Accaim Pep Map 100, 75 μm × 2 cm, nano Viper C18, 3 μm, Thermo Fischer Scientific, USA). The mobile phases, A and B, were used containing 0 and 100% acetonitrile, respectively, each supplemented with 0.1% formic acid. The trap column desalted and concentrated the peptide samples at a flow rate of 10 μL min^−1^ for 10 min with buffer A followed by elution with buffer B with a 150 min, 0–50% linear gradient at a flow rate of 300 nL min^−1^. To avoid sample carryover, the column was washed with 10 column volumes of buffer B after elution followed by re-equilibration with buffer A. All MS/MS spectra were acquired for fragmentation of the five most abundant peaks in a data-dependent mode with 35% normalized collision energy. An electrospray voltage of 2.2 kV was used to enter the separate peptides into the mass spectrometer. The dynamic exclusion duration was 180 s with an exclusion mass width of 0.5 Da. A mass spectral range of 150–2000 m/z was used for acquiring MS spectra.

### Protein Identification and Quantification

The obtained MS/MS spectra of proteins were searched in the universal Protein Resource Database (http://www.uniprot.org) using Mascot Daemon (Version 2.4.1, Matrix Science, London, UK). Criteria for identification of peptides were based on monoisotopic mass selection, precursor mass tolerance of ± 1.5 Da, fragment mass tolerance of ± 0.8 Da, two missed trypsin cleavage, and fixed modification of carbamidomethyl cysteine. For each database search, the calculated threshold score based on the Mascot ion score threshold (0.5) was used for accepting the individual spectrum. The false discovery rates (FDR) for peptides and proteins were calculated by Peptide Validator at 1.0% above the identity threshold.

The label-free quantitation analysis using emPAI was performed according to Shinoda et al. (2010). Common contaminant and decoy matches were filtered from the protein identification list. For the determination of protein identification, at least two unique peptides per protein were required. Relative quantification was done for proteins detected in at least two technical replicates of three biological trials in each group. An arithmetic mean was used to obtain the average label-free quantification within each biological group and was statistically evaluated through a two-sided t-test. The calculated p-value was corrected using false discovery rate-based multiple hypothesis testing using Perseus statistics software. All data were normalized using linear regression analysis.

### Bioinformatics Analysis

Fold changes of the differentially abundant proteins (DAPs) were calculated by comparing the abundances among the treatment comparisons. Significantly more abundant and less abundant proteins were categorized using DAVID Bioinformatics Tools (https://david.ncifcrf.gov) based on biological processes, molecular functions, and cellular components. Various metabolic pathways were determined through the Kyoto Encyclopedia of Genes and Genomes (KEGG) database (http://www.genome.jp/kegg/pathway.html).

### Statistical Analysis and Visualization

The experiment was carried out in a completely randomized block design. Heatmaps were generated with the web-based tool *clustvis* (Metsalu and Vilo 2015). Venn diagrams were visualized using the web-based tool Venny (Oliveros 2015).

## Results

### Systemic Endophytic Colonization of *M. oryzae* CBMB20

**Fig. 1:**
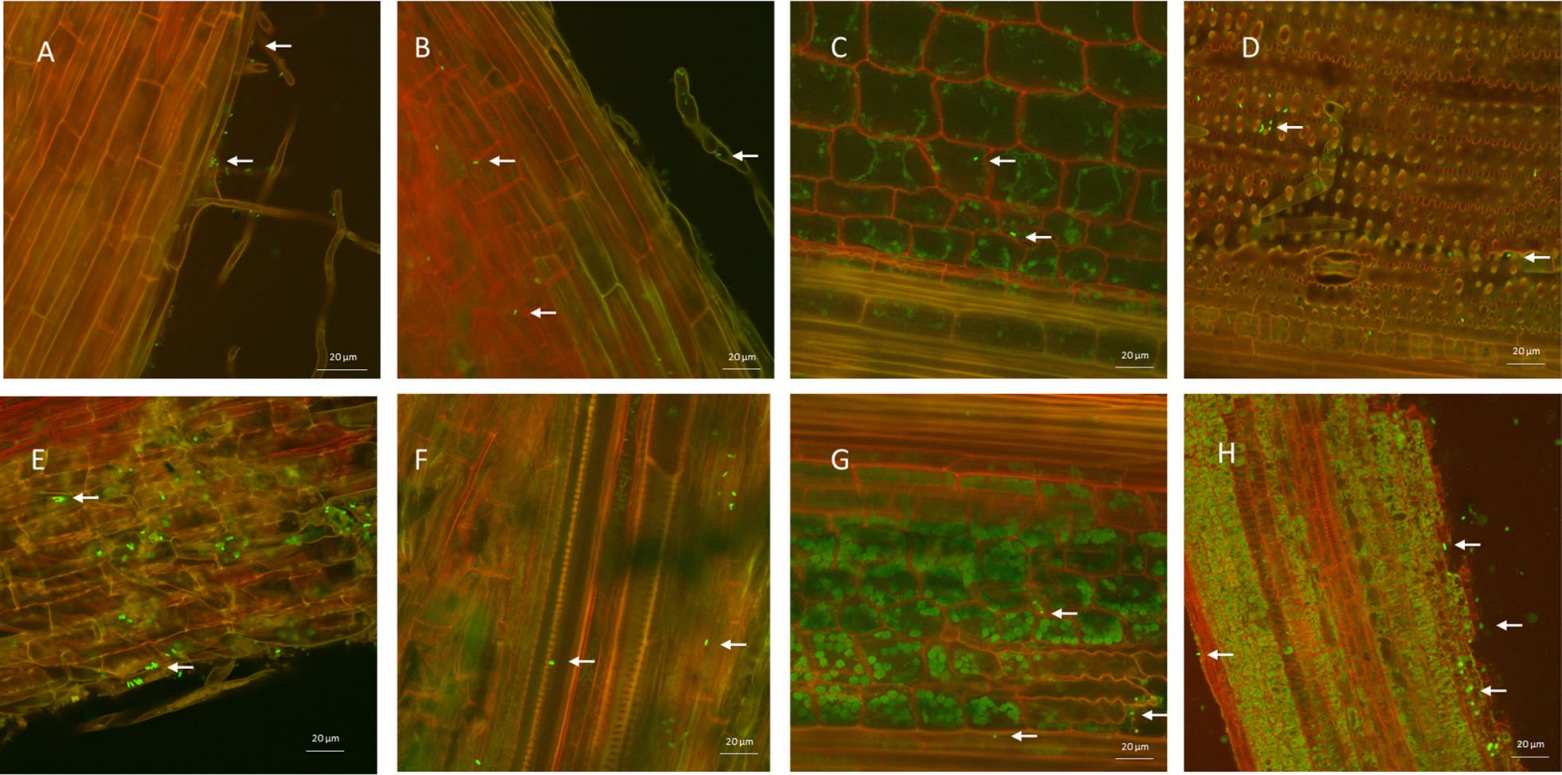
Colonization of *Methylobacterium oryzae* CBMB20-gfp in IR29 (top plane) and FL478 (bottom plane) root **A**, **B**, **E**, **F** stem (**C**, **G**) and leaf (**D**, **H**)

Root, stem, and leaf samples of IR29 and FL478 were prepared after 7 days post inoculation for confocal microscopy. The CBMB20-gfp tagged cells could be easily distinguished as green fluorescing bacteria in most of the samples prepared even with high background fluorescence. CBMB20 could be seen to persist in the root rhizosphere occupying the root hair junction and on the surfaces of the root hairs (Fig. 1A, B). Endophytic colonization was successful for both IR29 (Fig. 1B, D) and for FL478 (Fig. 1F, H). Systemic endophytic colonization was observed as CBMB20 internally colonized the roots (Fig. 1B, F), stems (Fig. 1C, G), and leaves (1D, 1H). Phyllospheric colonization could also be observed as few cells were detected on the leaf surface of FL478 (Fig. 1H). Specific internal colonization could be observed in the intercellular regions (Fig. 1B, D, G, H) and xylem elements (1F).

### Overall Proteomics of IR29 and FL478

**Fig. 2:**
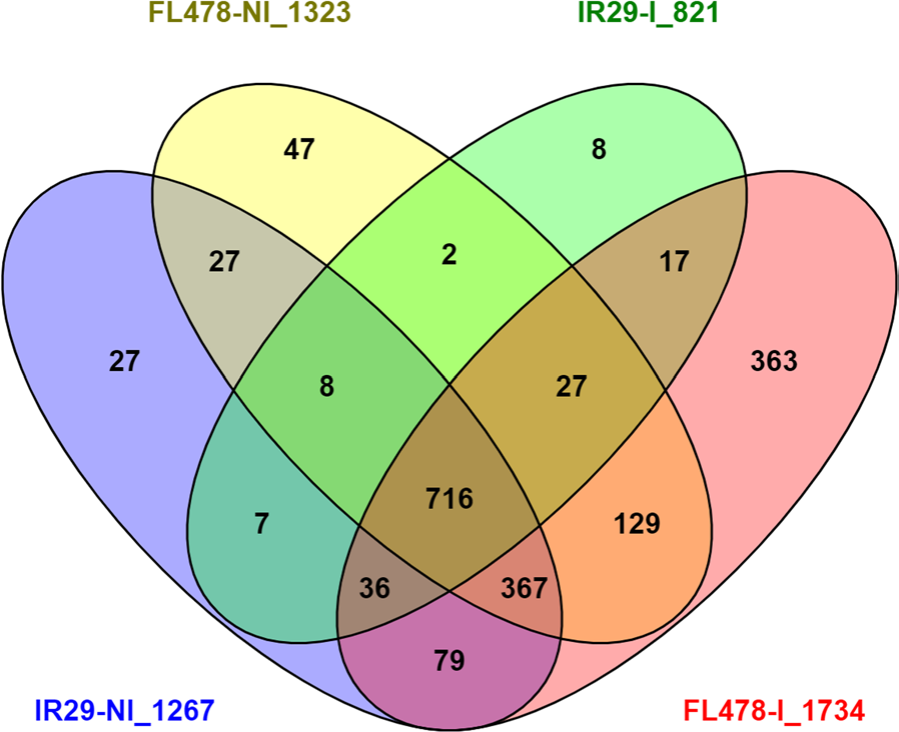
Venn diagrams of total identified, common and unique proteins in two rice genotypes, IR29 and FL478 under non-inoculation and *Methylobacterium oryzae* CBMB20 inoculation grown under normal greenhouse conditions. Numbers after the treatment name is the total number of proteins identified in the treatment present in all three replicates

**Fig. 3:**
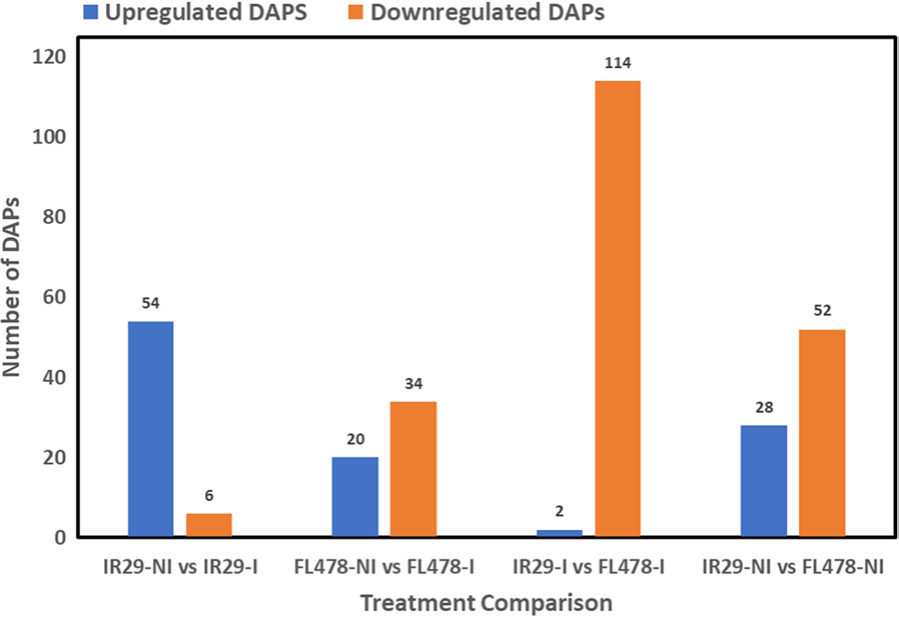
The total number of upregulated and downregulated differentially abundant proteins (DAPs) among the different treatment comparisons

Through a gel-free quantitative proteomics approach, a total of 3908 proteins were detected in rice plants across all the treatments at a 95% confidence level. A total of 1267, 1323, 821, and 1734 proteins are found in IR29-NI, FL478-NI, IR29-I, and FL478-I, respectively (Fig. 2). The number of common proteins found in all treatments range from 41 to 87% relative to their individual total protein. However, the similarity between the non-inoculated IR29 (IR29-NI) and FL478 (FL478-NI) is around 84–88%. Differentially abundant proteins (DAPs) could be observed when comparing treatments. It was observed that the abundances of 28 and 52 proteins were significantly increased and decreased, respectively, in IR29-NI versus FL478-NI treatment comparison. On the other hand, 54 and 6 proteins, 20 and 34 proteins, and 2 and 114 proteins were increased and decreased in IR29-NI versus IR29-I, FL478-I versus FL478-I, and IR29-I versus FL478-I treatment comparisons, respectively (Fig. 3, Additional file 2. Table S1) where unique and common significant DAPs between treatment comparisons also occur (Additional file 1. Fig. S1).

### Gene Ontology (GO) Terms of Proteomes in IR29 and FL478 During the Early Seedling Development

**Fig. 4:**
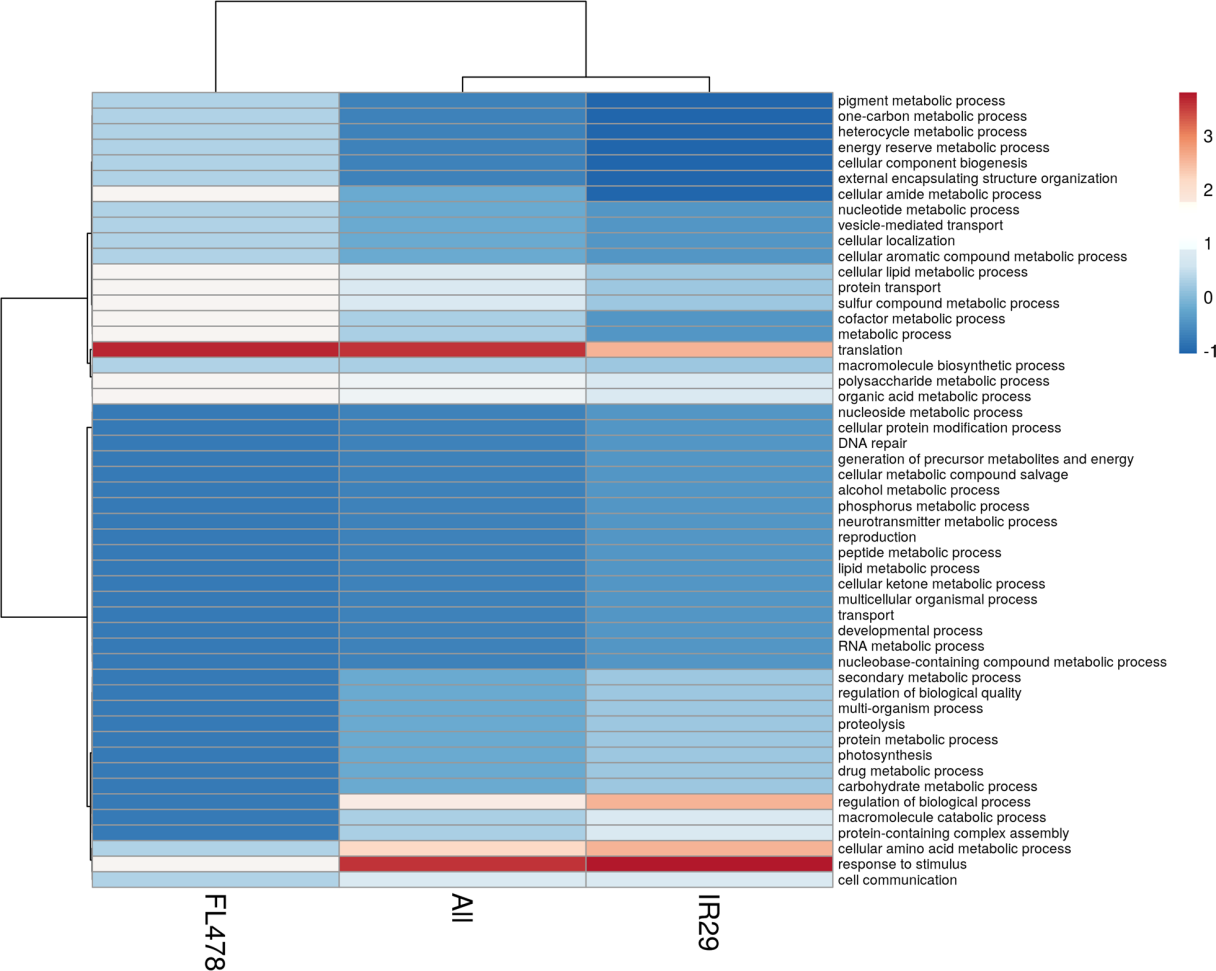
Gene ontology terms of differentially abundant proteins in IR29-NI compared to FL479-NI based on biological process observed during the 14th day seedling development grown under normal greenhouse conditions

**Fig. 5:**
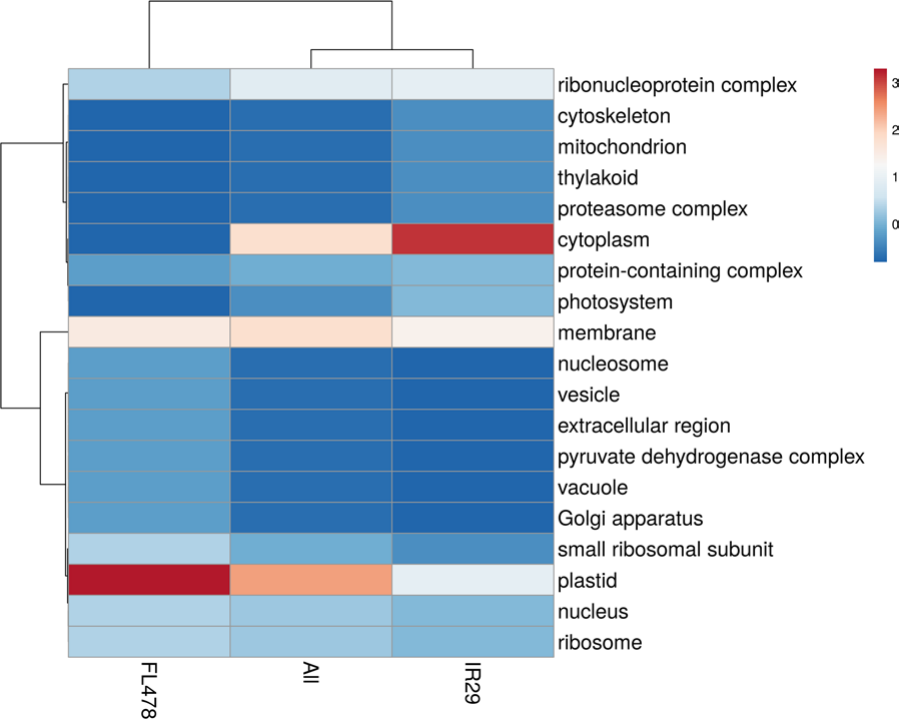
Gene ontology terms of differentially abundant proteins in IR29-NI compared to FL479-NI based on cellular component observed during the 14th day seedling development grown under normal greenhouse conditions

**Fig. 6:**
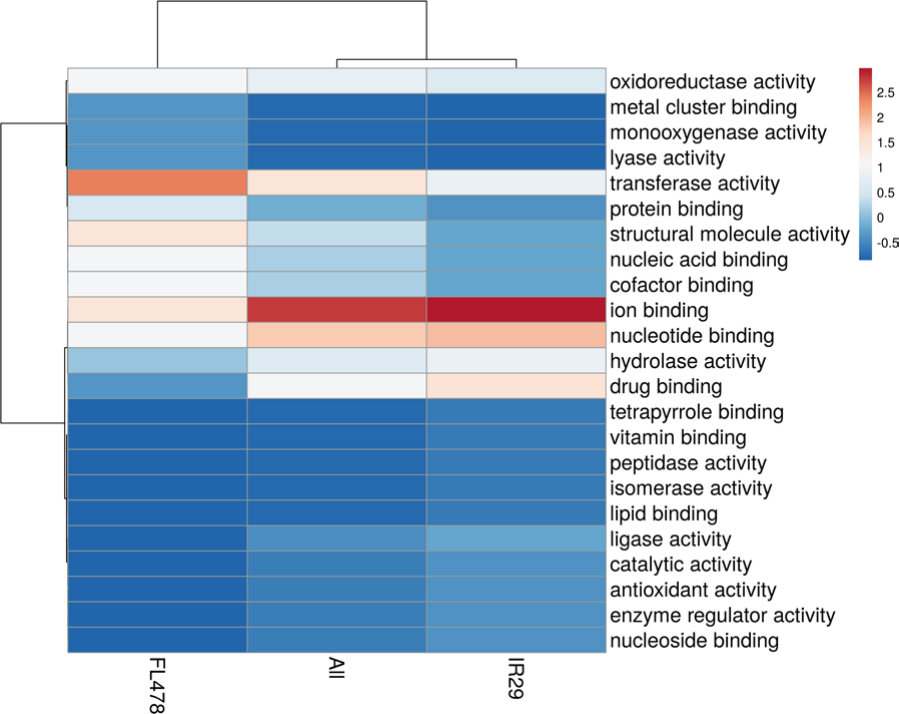
Gene ontology terms of differentially abundant proteins in IR29-NI compared to FL479-NI based on molecular function observed during the 14th day seedling development grown under normal greenhouse conditions

The comparison of the IR29-NI and the FL478-NI could infer inherent phenotypic differences between the two contrasting rice genotypes in terms of their expressed proteomes during the rapid early seedling developmental stages. The GO enrichment-based cluster analysis of the more abundant and less abundant proteins was determined in terms of biological process (Fig. 4; Additional file 3. Table S2), cellular component (Fig. 5; Additional file 3. Table S2), and molecular function (Fig. 6; Additional file 3. Table S2). The majority of the DAPs that are more abundant in IR29 belonged to response to stimulus (9.09%), regulation of biological process (6.82%), translation (6.82%), and cellular amino acid metabolic process (6.82%) in the biological process category (Fig. 4). These DAPs are mainly associated to the cytoplasm (25.71%), membrane (14.29%), plastid (11.42%) and the ribonucleoprotein complex (11.43%) (Fig. 5) carrying out the molecular functions for ion binding (19.57%) and nucleotide binding (14.13%) (Fig. 6, Additional file 4. Table S3). On the other hand, the majority of the DAPs that are more abundant in FL478 clustered in translation (11.43%) followed by less but equally abundant proteins in the biological process category (Fig. 4). These proteins are mainly localized in the plastids (26.92%) and membranes (15.38%) associated to molecular functions mainly transferase activity (16.67%), ion binding (11.9%) and structural molecule activity (11.9%) (Fig. 5, Fig. 6, Additional file 5. Table S4).

### Gene ontology (GO) Terms of Proteomes in IR29 and FL478 During *M. oryzae* CBMB20 Inoculation

**Fig. 7:**
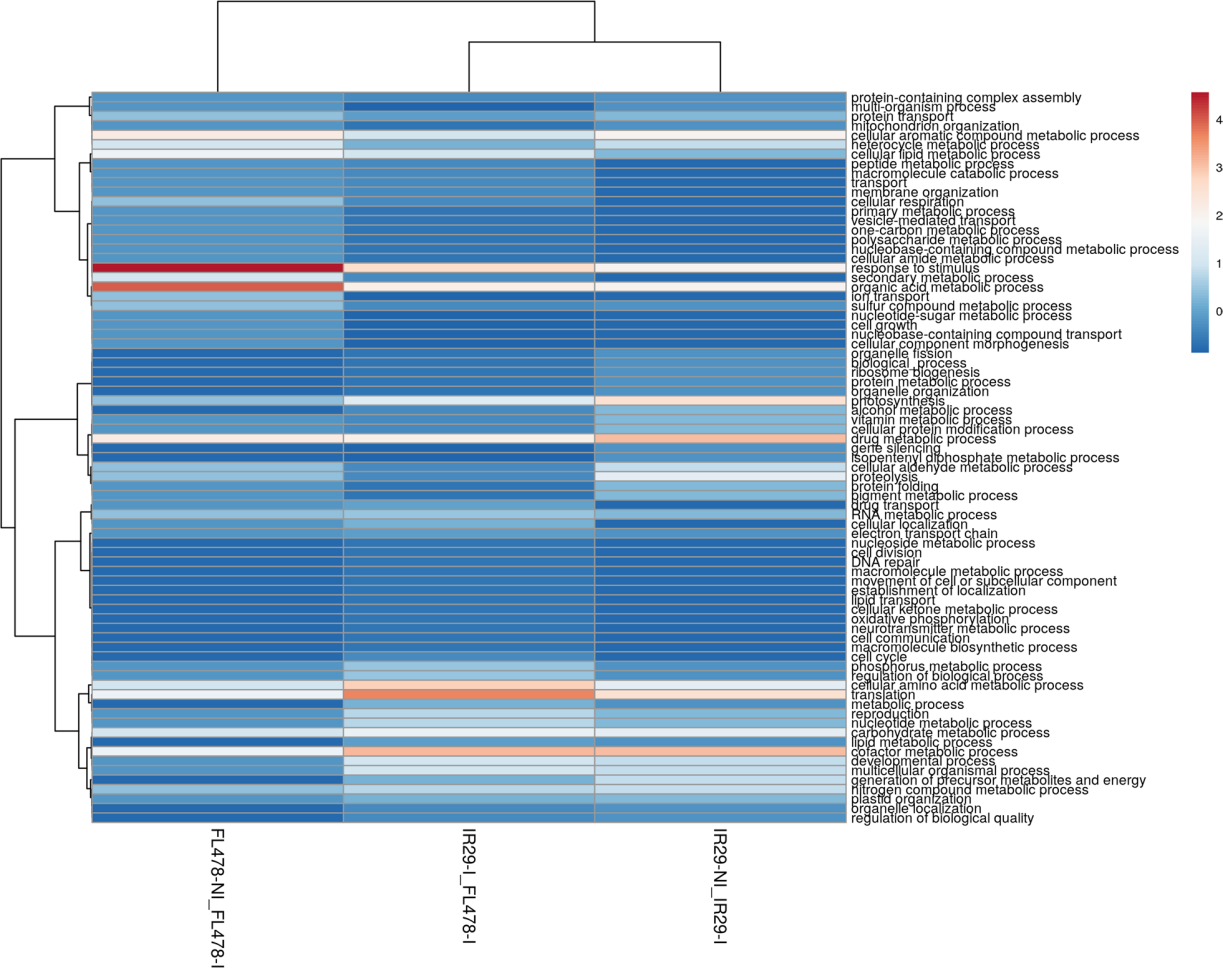
Gene ontology terms of differentially abundant proteins in different treatment comparisons based on biological process observed due to inoculation of *Methylobacterium oryzae* CBMB20

**Fig. 8:**
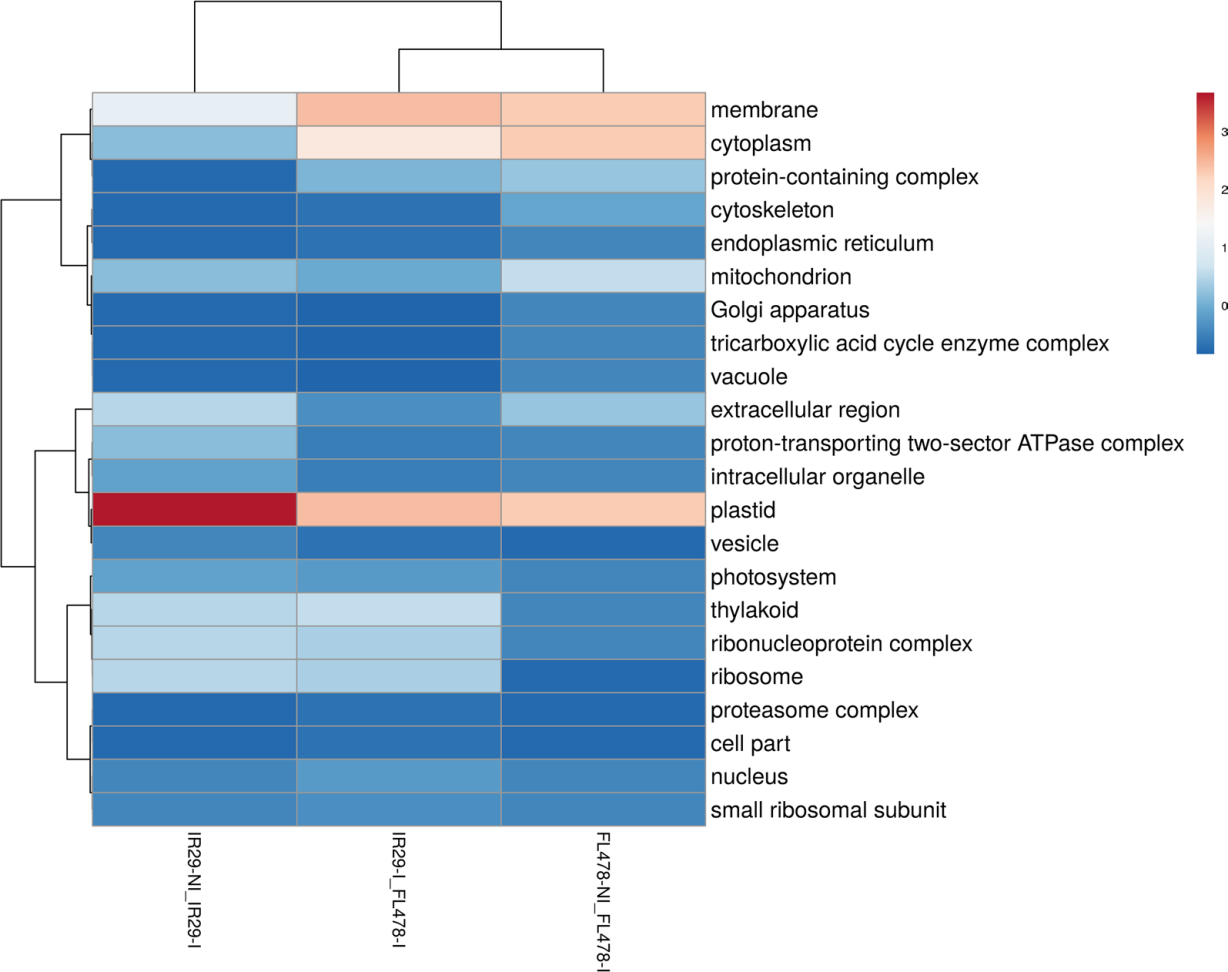
Gene ontology terms of differentially abundant proteins in different treatment comparisons based on cellular compartmentalization observed due to inoculation of *Methylobacterium oryzae* CBMB20

**Fig. 9:**
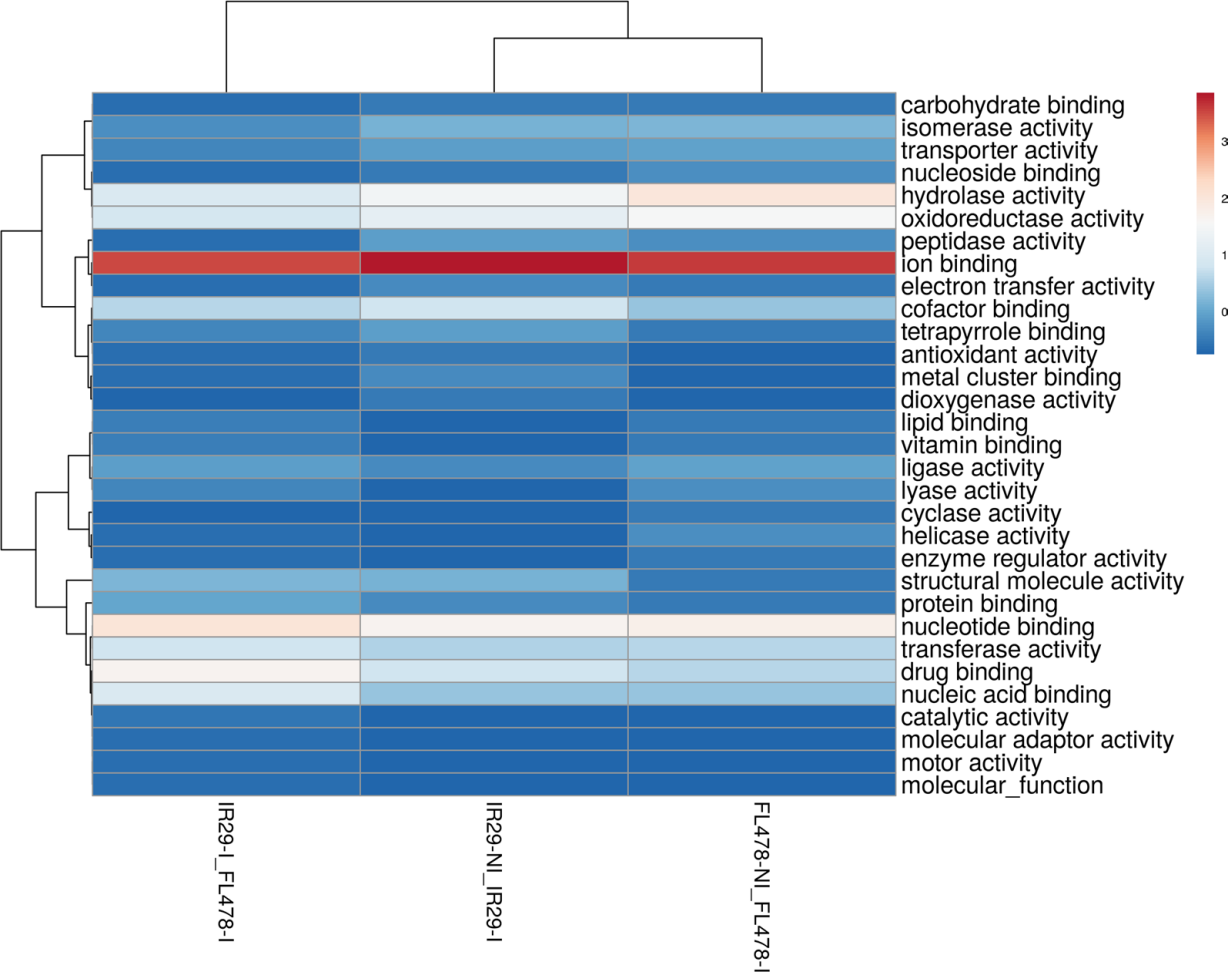
Gene ontology terms of differentially abundant proteins in different treatment comparisons based on molecular function observed due to inoculation of *Methylobacterium oryzae* CBMB20

Candidate DAPs in rice changing in abundance due to *M. oryzae* CBMB20 interactions could be inferred by comparing the IR29-NI vs. IR29-I and FL478-NI versus FL478-I. Additional information could also be derived when comparing IR29-I and FL478-I (Additional file 6. Table S5, Additional file 7. Table S6, Additional file 8. Table S7). The GO enrichment-based cluster analysis for biological process (Fig. 7), cellular component (Fig. 8), and molecular function (Fig. 9) of significantly abundant DAPs show potential rice genotype individual responses, similarities, and differences when interacting with CBMB20. The GO terms of DAPs for biological process in IR29 shifts in abundance from response to stimulus, cellular amino acid metabolic process, regulation of biological process and translation to cofactor metabolic process (6.31%), translation (5.41%) and photosynthesis (5.41%). The rice genotype FL478 showed a different shift in protein abundance from translation related to response to stimulus (9%) and organic acid metabolic acid (8%) (Fig. 7). The cellular components to which these DAPs are associated also shifts from cytoplasm-related to plastid (26.92%) and membrane (11.53%) for IR29 and from plastid-associated to a more balanced plastid (18%), membrane (18%), and cytoplasm-associated (18%) proteins in FL478 (Fig. 8). These DAPs in IR29 and FL478 are mainly carrying the molecular function of ion binding, nucleotide binding, hydrolase activity, and oxidoreductase activity (Fig. 9). In general, rice-*M. oryzae* CBMB20 interactions lead to shifts in the abundance of DAPs, quite distinct for both IR29 and FL478. It is also noteworthy to emphasize that there was an expansion of GO terms of the biological process and molecular functions due to inoculation of CBMB20, although the relative abundances of proteins associated with these GO terms were generally much lower.

### Proteomes Responsive to *M. oryzae* CBMB20 inoculation in IR29 and FL478

**Fig. 10:**
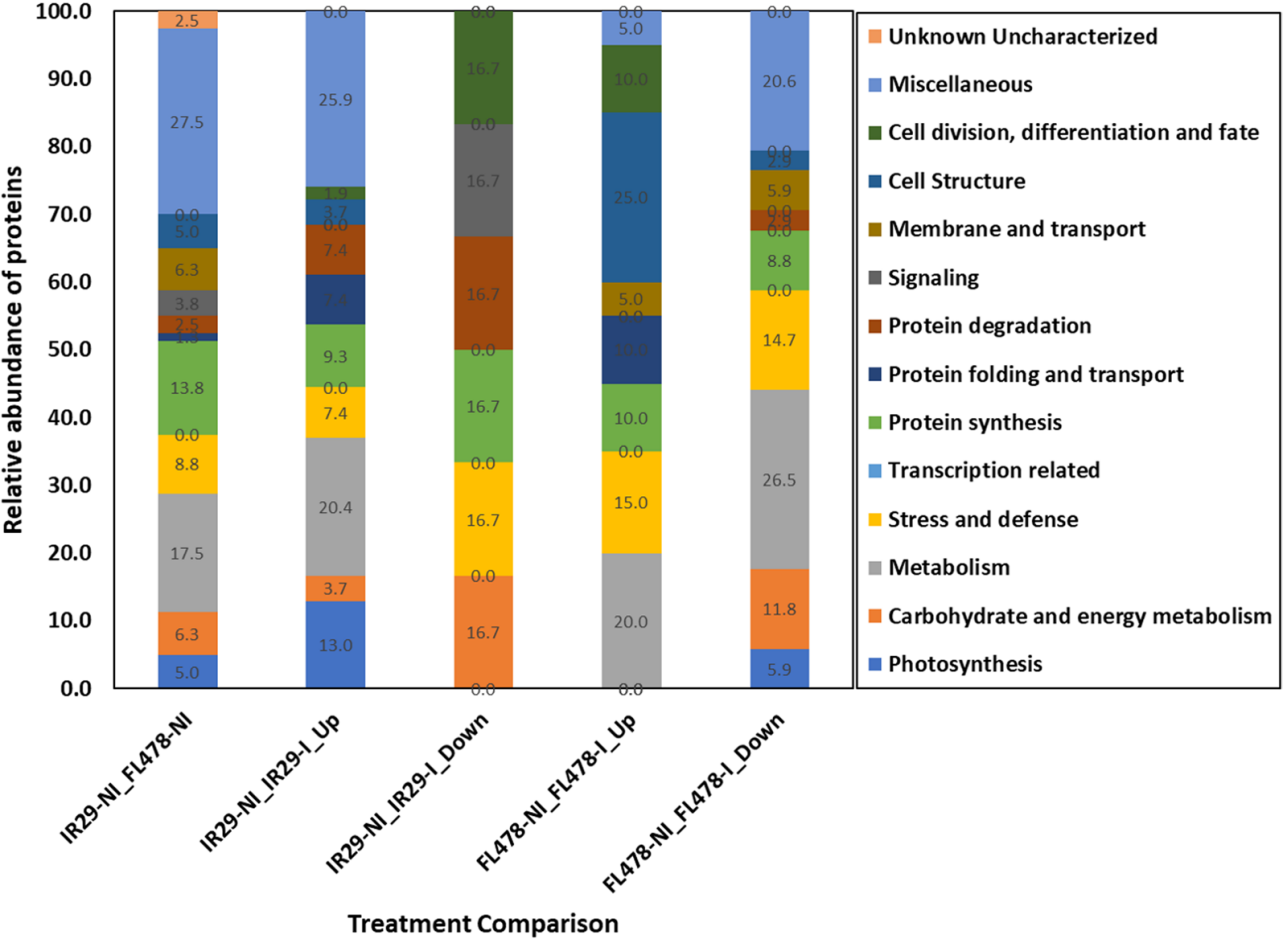
Relative abundance of DAPs upregulated or downregulated upon *Methylobacterium oryzae* CBMB20 inoculation categorized in terms of their main biological function and process involved in

The significantly abundant proteomes in IR29 and FL478 consisting of 60 and 54 DAPs, respectively, are associated with major functions and processes contributing to overall plant growth and development. Specific proteins associated with photosynthesis and metabolism were more prominently abundant in IR29 (Fig. 10) while specific proteins associated with protein folding and transport, membrane and transport, cell structure, and cell division, differentiation, and fate were more distinctly abundant in FL478 (Fig. 10; Additional file 6. Table S5). Out of 54 significantly abundant DAPS in IR29, seven are mainly photosynthesis-related proteins including NADPH-protochlorophyllide oxidoreductase (A2XZO1), protein THYLAKOID FORMATION1 (A2YML2), ATP synthase subunit b, chloroplastic (P0C2Y9), photosystem I P700 chlorophyll a apoprotein (P0C3557), photosystem I iron-sulfur center (P0C360), protein RETICULATA-RELATED 1, chloroplastic-like (A2ZJL3), and thylakoid luminal 16.5 kDa protein, chloroplastic (B8B267). These photosynthesis-related proteins are mainly associated with the thylakoid membrane of the plastid. There are also significantly abundant DAPs in IR29 involved in numerous metabolic processes and protein synthesis. On the other hand, significantly abundant DAPs in FL478 are associated with metabolism and protein synthesis as well as cell structure, organization, differentiation, and fate including outer envelope protein 80 (A2X208), oxalate—CoA ligase (A2XZ41), V-type proton ATPase subunit C (B8AXG9), and mitochondrial import receptor subunit TOM20 (A2WYG9) (Fig. 10, Additional file 7. Table S6).

A few patterns could also be observed when assessing effects of CBMB20 on the proteomes of IR29 and FL478. More notably, there are similar proteins that were upregulated in both IR29 and FL478 upon CBMB20 inoculation. Enzymes peptidyl-prolyl cis–trans isomerase (A2WJU9), thiamine thiazole synthase (A2YM28), and alanine—tRNA ligase (B8B4H5) were all upregulated in CBMB20-inoculated IR29 and FL478 (Table 1; Additional file 1: Fig. S1). There are also proteins and enzymes with similar functional categories downregulated due to CBMB20 inoculation. In FL478 observed in both FL478-NI versus FL478-I and IR29-I versus FL478-I treatment comparisons, thioredoxin H-type (A2YIW7), glutathione transferase (A2Z9L8), enoyl reductase (B8B9S9), oxysterol-binding protein-related protein (A2XF220) and cytochrome b6 were lower in abundance (Table 2) associated to stress and defense and signaling. Although there are only six downregulated DAPs in IR29, the protein thioredoxin domain-containing protein, a stress-related protein was also similarly observed.

**Table 1:** Common upregulated DAPS between IR29 and FL478 due to CBMB20 inoculation

Upregulated DAPs in IR29 due to inoculation of CBMB20	Upregulated DAPs in FL478 due to inoculation of CBMB20	Protein function
A2WJU9 Peptidyl-prolyl cis–trans isomerase	A2WJU9 Peptidyl-prolyl cis–trans isomerase; (Short = PPIase; EC = 5.2.1.8)	0006464: cellular protein modification process
A2YM28 Thiamine thiazole synthase, chloroplastic; (AltName: Thiazole biosynthetic enzyme)	A2YM28 Thiamine thiazole synthase, chloroplastic; (AltName: Full = Thiazole biosynthetic enzyme; EC = 2.4.2.60)	0050896: response to stimulus; 0006725: cellular aromatic compound metabolic process; 0006766: vitamin metabolic process; 0006790: sulfur compound metabolic process; 0006807: nitrogen compound metabolic process; 0017144: drug metabolic process; 0046483: heterocycle metabolic process
B8B4H5 Alanine—tRNA ligase, chloroplastic/mitochondrial precursor	B8B4H5 Alanine—tRNA ligase, chloroplastic/mitochondrial precursor; (EC = 6.1.1.7; AltName: Full = Alanyl-tRNA synthetase; Short = AlaRS)	0006412: translation; 0006520: cellular amino acid metabolic process; 0016070: RNA metabolic process

**Table 2:** Downregulated DAPS in FL478 due to *Methylobacterium oryzae* CBMB20 inoculation

Downregulated DAPs in FL478 due to inoculation of CBMB20 (Protein Accession Number)	Downregulated DAPs in FL478 due to inoculation of CBMB20 (Protein Name)	Protein function
B8B9S9	Enoyl-[acyl-carrier-protein] reductase [NADH] 1, chloroplastic	0006082: organic acid metabolic process; 0044255: cellular lipid metabolic process
A2YIW7	Thioredoxin H-type; (Short = Trx-H; AltName: Full = Phloem sap 13 kDa protein 1)	0006810: transport; 0050896: response to stimulus
A2Z9L8	Glutathione transferase; (EC = 2.5.1.18)	0006518: peptide metabolic process; 0006790: sulfur compound metabolic process; 0051186: cofactor metabolic process; 0050896: response to stimulus
P0C316	Cytochrome b6	0015979: photosynthesis; 0022900: electron transport chain; 0045333: cellular respiration
A2XF22	Oxysterol-binding protein-related protein 3B	0008289: lipid binding

## Discussion

The current study was conducted to determine how bacterial inoculation with a multifunctional plant growth promoting *M. oryzae* CBMB20 affects the expression of proteins in rice plants, IR29 and FL478, and promotes plant growth and development during the rapid changes occurring during the early seedling development. We initially observed the endophytic colonization of CBMB20 in IR29 and FL478. To establish inherent phenotypic proteomic differences between IR29 and FL478, IR29-NI and FL478-NI were compared. To assess microbe-mediated proteomic changes due to CBMB20 inoculation, treatment comparisons between IR29-NI vs. IR29-I and FL478-NI versus FL478-I were analyzed. Additional information could also be derived in the inoculated plants by comparing IR29-I versus FL478-I.

### Inherent Phenotypic Similarities and Differences Between IR29 and FL478 Expressed Through Their Proteomes

The cultivars IR29 and FL478 are genotypically closely related rice cultivars where FL478 traces its parental lineage to IR29 and Pokkali of which IR29 is the high-yielding salt-sensitive parent and Pokkali is the salt-tolerant low-yielding parent (Walitang et al. 2019). The present study validates the close relatedness of the two rice genotypes as almost 88% of their phenotypically expressed proteins during their early seedling development are exactly similar. However, the level of expression of these proteins potentially differs between the two rice cultivars as we observed 28 DAPs in the salt-tolerant FL478 compared to 52 DAPs in the sensitive rice variety IR29. The remaining differences in expressed proteins may further lead to distinct differences in IR29 and FL478. The differences in certain biological functions and processes observed in this study corroborated the transcriptional and translational differences between the parent genotypes observed in an earlier study (Li et al. 2018).

Overall, the current study shows that a majority of the DAPs associated with FL478 and IR29 are related to metabolism, protein synthesis, stress and defense, carbohydrate and energy metabolism, membrane, and transport. However, earlier studies show inherent differences between the two rice genotypes become apparent especially under salt stress conditions in which proteomes and transcripts point to variations in salt-stress adaptive defenses between the two rice cultivars. Previous comparative studies under salt stress between the two rice genotypes showed that FL478 shows greater abundance and upregulation of genes and proteins associated with superoxide dismutase, peroxidase genes, fiber protein, and inorganic pyrophosphatase with concurrent higher relative growth rate and lower Na^+^/K^+^ in the roots (Hosseini et al. 2015; Senadheera et al. 2012). Differences in the biological process as well as molecular function were also observed between IR29 and FL478 through their transcriptional profile (Mirdar Mansuri et al. 2019) which corroborates the observed differences in terms of proteomics in the present study even under normal conditions. The observed differences between the two rice genotypes under normal growing conditions could be mainly attributed to the differences in gene expression profiles, both transcriptome and translatome, as seen in the comparison between IR29 and FL478’s paternal parent, Pokkali, showing distinct differences between the two even without salt stress (Li et al. 2018). Under normal conditions, Li et al. (2018) observed that the gene ontology terms overrepresented in Pokkali include regulation of metabolism, gene transcription, cellular carbohydrate metabolism, and DNA conformation while photosynthesis, response to stress, cell wall macromolecule metabolism and organization, transport and aminoglycan metabolism were observed for IR29. This pattern is quite similar to the proteomes of IR29 and FL478, especially after inoculation with CBMB20.

### Changes in the Functional Proteomes’ Gene Ontology Terms in IR29 and FL478 Induced by Inoculation of the Multi-faceted *Methylobacterium oryzae* CBMB20

*Methylobacterium oryzae* CBMB20 has been well-documented in enhancing plant growth under normal (Lee et al. 2006; Ryu et al. 2006; Madhaiyan et al. 2010) and stress conditions (Indiragandhi et al. 2008; Yim et al. 2013, 2014; Chanratana et al. 2018; Chatterjee et al. 2019). Common features of *M. oryzae* CBMB20 plant growth promotion usually include enhanced germination, germination rate, and seedling vigor index together with an increased shoot and root length and increased biomass. However, there are potential differences in the responses across crop plants due to the inoculation of CBMB20 (Ryu et al. 2006; Madhaiyan et al. 2010). In the current study, distinct differences in cultivar responses even at the proteome level could be observed between IR29 and FL478. Interestingly, inoculation with CBMB20 expanded the gene ontology terms of the different proteins in both IR29 and FL478. The proteomes of IR29 and FL478 presented a much more diversified gene ontology terms upon inoculation with CBMB20. This supports the idea that inoculation with CBMB20 has extended the phenotype of the rice plants (Dawkins 1999; Hardoim et al. 2015) mediated by the microbe-responsive proteomes.

Comparing the functional categories of the proteins associated with the CBMB20 inoculated IR29 and FL478 shows that there are more proteins related to photosynthesis, metabolism, and protein degradation in IR29 while more proteins are associated with metabolism, stress and defense, cell structure, differentiation and fate, and protein folding and transport were observed in FL478. Roy Choudhury et al. (2022) showed that under normal growing conditions, inoculation with CBMB20 of a rice *japonica* cultivar increased the chlorophyll contents and carotenoid content of the leaves. In the present study, a protein directly involved with pigment synthesis (NADPH-protochlorophyllide oxidoreductase—A2XZ01) and chlorophyll-carotenoid formation was upregulated in IR29. Other photosynthesis-related proteins detected are mainly involved in the integrity and function of the chloroplast. The upregulated proteomes of IR29 and FL478 due to CBMB20 seem to reflect the enriched functional categories of transcriptomes and translatomes in IR29 and Pokkali (Li et al. 2018) and may point to a notion that CBMB20 inoculation reinforces these inherent differences in the two genotypes. Earlier, metabolomic and transcriptomic changes in *Arabidopsis* inoculated by *Pseudomonas fluorescens* SS101 have been established to be a strain-specific response (van de Mortel et al. 2012). However, our study showed that there are also plant genotype-specific proteomic responses occurring due to inoculation such as the enrichment of photosynthesis-related proteins in IR29 and proteins related to cell differentiation in FL478. Although CBMB20 is an efficient plant growth promoter in multiple crop plants, the observed plant genotype-specific responses could explain differences in the effectiveness of bioinoculants and the need to investigate the compatibility of crop and microbial inoculants such as those observed under field conditions (Sanjenbam et al. 2022).

### Common Microbe-Responsive Proteins Modulated by *Methylobacterium oryzae* CBMB20

Although there is a difference in the overall response of IR29 and FL478 to CBMB20 inoculation, there are similar proteins upregulated indicating similar modes of microbe-mediated proteomic changes in both rice genotypes. There were three proteins both upregulated in IR29 and FL478 upon CBMB20 inoculation viz peptidyl-prolyl cis–trans isomerase (A2WJU9), thiamine thiazole synthase, chloroplastic (A2YM28), and alanine—tRNA ligase, chloroplastic/mitochondrial precursor (B8B4H5).

The enzyme peptidyl-prolyl cis–trans isomerase catalyzes the isomerization between the cis and trans form of peptide bonds (Lin et al. 2019) particularly certain proline residues involved in protein folding (Blecher et al. 1996) and cellular protein modification process (GO: 006464). This enzyme is also regulated by various environmental stresses and could act as a molecular timer in diverse plant physiological and pathological processes (Blecher et al. 1996). Thiamine thiazole synthase is essential for thiamine biosynthesis especially targeted to chloroplasts influencing chloroplast development (Feng et al. 2019). However, the thiamine thiazole synthase detected in IR29 and FL478 is also related to response to stimulus (GO: 0050896) and different metabolic processes (GO: 0006727—aromatic compound, GO: 0,006,777 – vitamin, GO: 0006790—sulfur compound, GO: 0006807—nitrogen compound, and GO: 0046483—heterocycle metabolic process). In addition, based on gene ontology, alanine—tRNA ligase chloroplastic/mitochondrial precursor (B8B4H5), which attaches alanine to tRNA, is associated with translation (GO: 0006412), cellular amino acid metabolic process (GO: 0016070), and RNA metabolic process (GO: 0,016,070). The upregulation or enrichment of exactly similar proteins or proteomes with analogous functions in the two rice genotypes is potentially due to the same responses brought about by CBMB20 producing metabolites and proteins involved with plant growth promotion including hormones (Lee et al. 2006; Ryu et al. 2006; Madhaiyan et al. 2010) and improvement of plant nutrient contents (Anandham et al. 2007; Madhaiyan et al. 2010; Kwak et al. 2014).

*Methylobacterium oryzae* CBMB20 has been also observed to induce defense responses and was able to increase resistance to subsequent biotic stresses indirectly promoting growth through plant health (Indiraghandi et al. 2008; Yim et al. 2013; Yim et al. 2014; Roy Choudhury et al. 2023). Colonization of non-native bacterial endophytes usually results in the activation of the plant’s defense responses and the colonizer has to either evade or modulate these responses in order to beneficially coexist with their host (Pieterse et al. 2014; Balmer et al. 2015). Several defense-related proteins have been modulated after rice inoculation with CBMB20. In FL478, thioredoxin H-type (A2YIW7) and glutathione transferase (A2Z9L8) were repressed, similar to the thioredoxin domain-containing protein (A2YL83) observed in IR29. These proteins are important for regulating redox reactions and redox-mediated defense signaling (Yoo et al. 2011; Hosseini et al. 2015; Chi et al. 2019). These stress-responsive proteins are potentially regulated indirectly through the reduction of stress ethylene (Indiraghandi et al. 2008; Yim et al. 2013; Yim et al. 2014; Roy Choudhury et al. 2023). These results show that CBMB20 has the capacity to modulate defense responses in rice and is different from those observed in pathogenic bacteria (Pieterse et al. 2014; Balmer et al. 2015) where their presence lead to hypersensitive response or induction of systemic acquired resistance aimed at eliminating the invading pathogens. The modulation of stress responses by CBMB20 suggests that rice-CBMB20 interactions contribute to the indirect mechanism of plant growth promotion as CBMB20 inoculation potentially results to tolerance enhancement when rice plant is exposed to biotic and abiotic stress.

## Conclusion

The interaction of rice and the plant growth-promoting bioinoculant, *Methylobacterium oryzae* CBMB20 leads to dynamic proteome responses potentially resulting in beneficial plant–microbe interactions. This study showed that the genotypically related rice cultivars IR29 and FL478 phenotypically express around 90% similar proteins during their early seedling developmental stages. There are common responsive proteins upon CBMB20 inoculation indicating similar mechanisms of plant growth promotion in IR29 and FL478, associated with the activation of vitamin biosynthesis related process and protein synthesis related process. However, there are distinct responses of IR29 and FL478 upon CBMB20 inoculation including a more pronounced enhancement of photosynthesis in IR29 and increased proteins related to cell structure, cell differentiation and fate, and protein folding and transport in FL478. *M. oryzae* CBMB20 inoculation also regulated stress and defense-related proteins resulting in the modulation of defense responses in rice upon bacterial inoculation. It would be interesting to see how responses in rice genotypes are linked to subsequent changes mediated by CBMB20 when rice hosts are exposed to biotic and abiotic stresses. In addition, integrated analysis of transcriptomes, proteomes, and metabolomes is crucial in elucidating the intricately complex plant–microbe associations.

## Supplementary Information

**Additional file 1. Fig. S1.** Venn diagrams of common and unique upregulatedand downregulated B DAPs due to Methylobacterium oryzae CBMB20 inoculation observed in different treatment comparisons.

**Additional file 2. Table S1.** List of significant differentially abundant proteins among treatment comparison.

**Additional file 3. Table S2.** Gene ontology terms of all DAPs in IR29-NI versus FL478-NI.

**Additional file 4. Table S3.** Gene ontology terms of more abundant DAPs in IR29-NI.

**Additional file 5. Table S4.** Gene ontology terms of more abundant DAPs in FL478-NI.

**Additional file 6. Table S5.** Gene ontology terms of significant DAPS in IR29-NI versus IR29-I.

**Additional file 7. Table S6.** Gene ontology terms of significant DAPs in FL478-NI versus FL478-I.

**Additional file 8. Table S7.** Gene ontology terms of significant DAPs in IR29-I versus FL478-I.

## Data Availability

The datasets supporting the conclusions of this article are included within the article and its additional files. The mass spectrometry data have been deposited to the MassIVE consortium  (https://massive.ucsd.edu/ProteoSAFe/static/massive.jsp) with dataset identifier MSV000091686.
