# Supplementary figures and images for: Microbe-Responsive Proteomes During Plant–Microbe Interactions Between Rice Genotypes and the Multifunctional Methylobacterium oryzae CBMB20
Source: Rice (N Y). 2023 May 5;16:23. doi: 10.1186/s12284-023-00639-y (PMC10163190; doi:10.1186/s12284-023-00639-y)

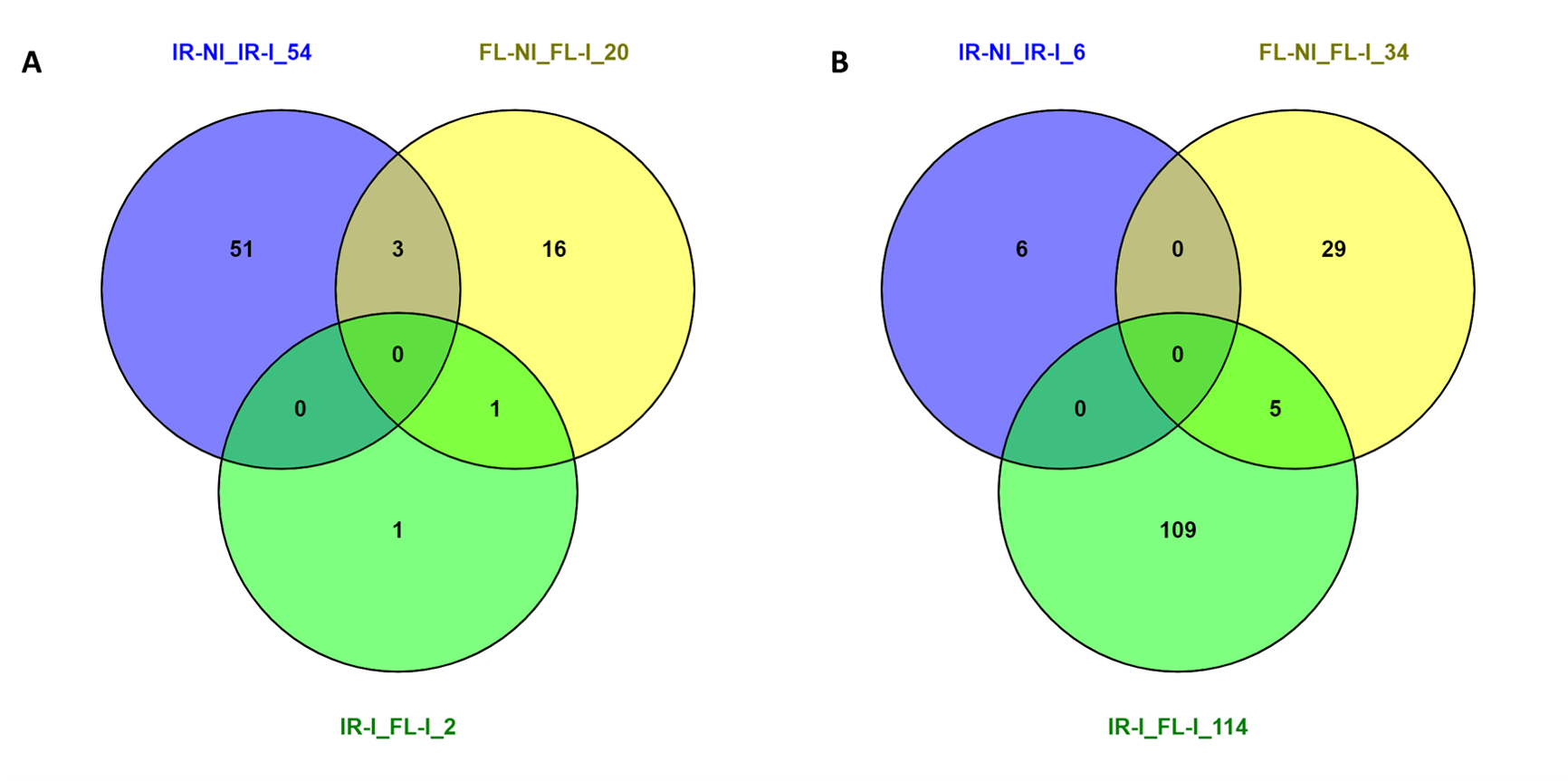

Supplement: Supplementary file 1 — Additional file 1. Fig. S1. Venn diagrams of common and unique upregulatedand downregulated B DAPs due to Methylobacterium oryzae CBMB20 inoculation observed in different treatment comparisons. [file 12284_2023_639_MOESM1_ESM.png]
